# Identification of *Creb3l4* as an essential negative regulator of adipogenesis

**DOI:** 10.1038/cddis.2014.490

**Published:** 2014-11-20

**Authors:** T-H Kim, S-H Jo, H Choi, J-M Park, M-Y Kim, H Nojima, J-W Kim, Y-H Ahn

**Affiliations:** 1 Department of Biochemistry and Molecular Biology, Yonsei University College of Medicine, Seoul 120-752, Republic of Korea; 2 Brain Korea 21 PLUS Project for Medical Sciences, Yonsei University College of Medicine, Seoul 120-752, Republic of Korea; 3 Department of Molecular Genetics, Research Institute for Microbial Diseases, Osaka University, Osaka, Japan

## Abstract

Understanding the molecular networks that regulate adipogenesis is crucial for combating obesity. However, the identity and molecular actions of negative regulators that regulate the early development of adipocytes remain poorly understood. In this study, we investigated the role of CREB3L4, a member of the CREB3-like family, in the regulation of adiposity. Constitutive overexpression of CREB3L4 resulted in the inhibition of adipocyte differentiation, whereas knockdown of *Creb3l4* expression caused differentiation of preadipocytes into mature adipocytes, bypassing the mitotic clonal expansion step. In 3T3-L1 preadipocytes, *Creb3l4* knockdown resulted in increased expression of peroxisome proliferator-activated receptor *γ* (PPAR*γ*2) and CCAAT/enhancer binding protein (C/EBP*α*), either by increasing the protein stability of C/EBP*β* or by decreasing the expression of GATA3, a negative regulator of PPAR*γ*2 expression. Consequently, increased PPAR*γ*2 and C/EBP*α* levels induced adipocyte differentiation, even in the presence of minimal hormonal inducer. Thus, it can be speculated that CREB3L4 has a role as gatekeeper, inhibiting adipogenesis in 3T3-L1 preadipocytes. Moreover, adipocytes of *Creb3l4*-knockout mice showed hyperplasia caused by increased adipogenesis, and exhibited improved glucose tolerance and insulin sensitivity, as compared with littermate wild-type mice. These results raise the possibility that *Creb3l4* could be a useful therapeutic target in the fight against obesity and metabolic syndrome.

Obesity is a worldwide epidemic in both developed and developing countries. Due to excess food intake, adipose tissue enlarges, *via* either hyperplasia or hypertrophy.^1^ Increased adipose tissue is associated closely with the development of insulin resistance and is the principal risk factor in the development of type 2 diabetes mellitus.^2^ Thus, studying the biological networks and transcription factors controlling adipogenesis, including adipocyte differentiation, may facilitate greater understanding of the pathophysiology of obesity and type 2 diabetes.

Adipogenesis is a multi-step process requiring a cascade of transcription factors that regulate the expression of genes involved in lipogenesis, *β*-oxidation, and fatty acid uptake.^3^ Members of the CCAAT/enhancer binding family, such as C/EBP*α*, −*β*, and -*δ*, have key roles in adipogenesis.^4, 5^ C/EBP*β* and C/EBP*δ* directly upregulate the expression of C/EBP*α* and peroxisome proliferator-activated receptor *γ* (PPAR*γ*).^6^ Upon expression, PPAR*γ* and C/EBP*α* cooperate to express the full adipogenic program, including induction of additional transcription factors and suppression of growth-associated genes.^7^

Recently, endoplasmic reticulum (ER) membrane-bound bZIP domain-containing transcription factors, such as cyclic AMP-responsive element-binding protein 3(CREB3)/Luman/LZIP, CREB3-like 1 (CREB3L1)/OASIS, BBF2H7/CREB3L2, CREBH/CREB3L3, and CREB4/CREB3L4, were identified.^8^ These proteins share a region of high sequence homology with activating transcription factor 6 (ATF6), having a transmembrane (TM), a transcription-activation, and a bZIP domain. CREB3L4 (also called AIbZIP, Tisp40, or ATCE1) is expressed in human prostate tissue,^9^ and *Creb3l4* disruption resulted in abnormal epididymal sperm nuclei, ER stress, and activation of caspase 12, leading to apoptosis of meiotic/postmeiotic germ cells.^10^

Mouse *Creb3l4* generates CREB3L4*α* and CREB3L4*β*, which differ in their 55-residue N-terminal extension. Deletion of the TM domain of CREB3L4 results in retention of the protein in the nucleus.^11^ CREB3L4*β*, but not CREB3L4*α*, activates transcription of ER degradation-enhancing *α*-mannosidase-like protein (Edem), which enhances unfolded protein degradation, presumably by acting on the unfolded protein response element (UPRE).^11^ The role of CREB3L4 in ER stress is beginning to be understood, while its role in adipocyte differentiation and obesity is unknown.

In this study, we demonstrate a novel role of CREB3L4 in adipogenesis. We demonstrate that CREB3L4 acts as a negative regulator of adipogenesis through dual mechanisms. Silencing of *Creb3l4* potently increases adipocyte differentiation even in the presence of minimal hormonal inducer bypassing the mitotic clonal expansion (MCE) step, enhancing the PPAR*γ*2 expression through regulating protein stability of C/EBP*β* and GATA3 expression *in vitro*. Mice with deleted *Creb3l4* showed improved metabolic parameters due to the increase in numbers of fat cells, caused by increased adipogenesis. These results provide a biological framework for discovery of potential target genes, such as *Creb3l4*, in obesity treatment.

## Results

### CREB3L4 is regulated during adipocyte differentiation

**Figure 1:**
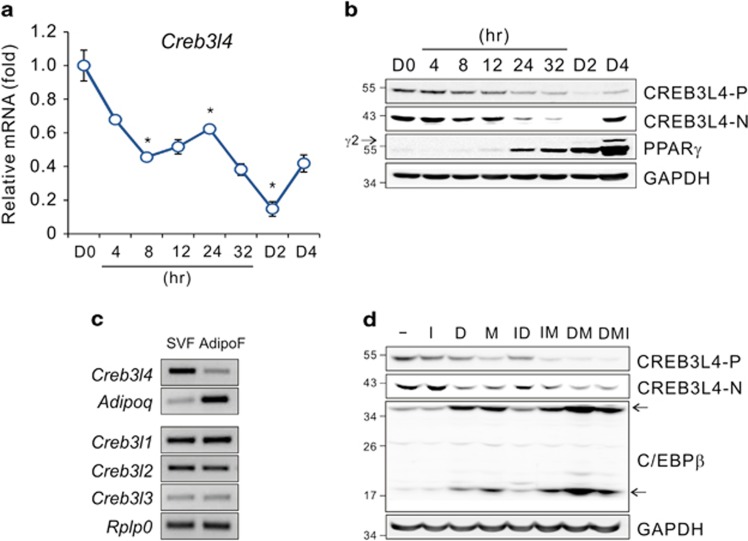
Regulation and function of CREB3L4 during adipocyte differentiation. (**a**) Real-time PCR analysis of *Creb3l4* mRNA levels in 3T3-L1 cells treated with differentiation cocktail (DMI: 0.5 mM 3-isobutyl-1-methylzanthine [IBMX; M], 1 *μ*M dexamethasone [D], and 1 *μ*g/ml of insulin [I]) (*n*=3). Error bars represent mean±S.E.M., *P<0.05. (**b**) Immunoblot of CREB3L4 during differentiation of 3T3-L1 preadipocytes, treated with DMI; CREB3L4-P, precursor form of CREB3L4; CREB3L4-N, nuclear form of CREB3L4. (**c**) Semi-quantitative RT-PCR analysis of the expression of the *Creb3l* family in the SVF and the AdipoF. SVF, stromal vascular fraction; AdipoF, adipose fat fraction. (**d**) Immunoblot of CREB3L4 and C/EBP*β* in 3T3-L1 cells treated for 1 day with individual components of the differentiation cocktail

To investigate whether the Creb-like family is involved in energy metabolism, we first examined the expression of the corresponding genes in various metabolic tissues of mice. *Creb3l3* is most abundantly expressed in the liver,^12^ but white adipose tissue (WAT) expressed markedly more *Creb3l4* as compared with other tissues suggesting that WAT could be one of the target tissue of CREB3L4 action (Supplementary Figure S1). The expression of *Creb3l4* in WAT led us to explore the role of CREB3L4 in adipocyte differentiation and the development of adiposity. Therefore, we wondered whether CREB3L4 is a regulatory component of the differentiation program, and whether its expression could be regulated during differentiation. During 3T3-L1 preadipocyte differentiation, *Creb3l4* expression decreased from day 0 (D0) and recovered after D2, as shown by real-time PCR (Figure 1a) and western blotting (Figure 1b). Fractionation of adipose tissue into mature adipocytes (AdipoF) and the stromal-vascular fraction (SVF) revealed that *Creb3l4* was more abundant in the SVF, which contains adipocyte precursors (Aps), than in AdipoF (Figure 1c). To determine which components of the differentiation cocktail were responsible for decreased *Creb3l4* expression, 3T3-L1 cells were stimulated for 1 day with insulin (I), dexamethasone (D), or isobutyl methylxanthine (M), alone, or in combination (DMI). Protein level of CREB3L4 was decreased the most by DMI treatment, followed by treatment with isobutyl methylxanthine, and with dexamethasone (Figure 1d). These results suggested that CREB3L4 may have a critical role in adipocyte differentiation.

### CREB3L4 acts as a negative regulator of adipogenesis

**Figure 2:**
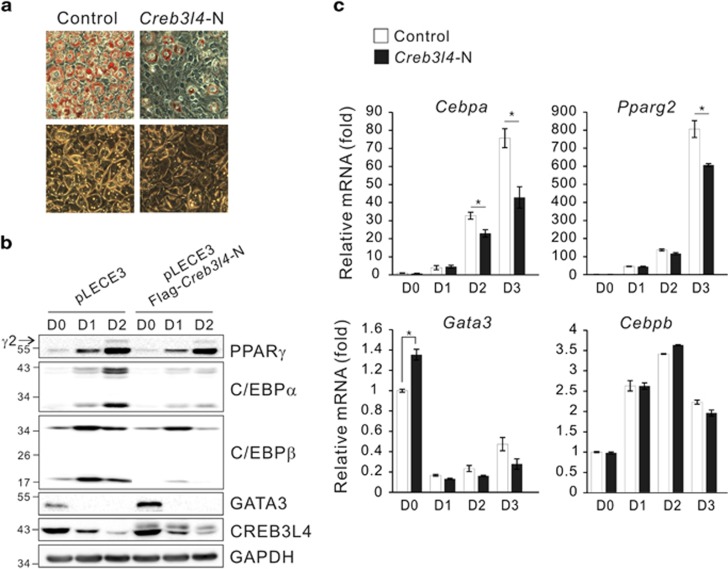
CREB3L4 inhibits the adipocyte differentiation. (**a**) 3T3-L1 cells were transfected with *Creb3l4* (N) by electroporation, after which differentiation was induced by differentiation cocktail (DMI: 0.5 mM 3-isobutyl-1-methylzanthine [IBMX; M], 1 *μ*M dexamethasone [D], and 1 *μ*g/ml of insulin [I]). Oil-red O staining of 3T3-L1 cells expressing *Creb3l4* (N) at day 8 (D8), and microscopy ( × 20) was performed. (**b**) 3T3-L1 cells were transduced with lentiviral Flag-*Cre3l4* (N) and harvested at the indicated times. Expression of critical adipogenic factors, as determined by immunoblot. (**c**) The mRNA levels of *Cebpa*, *Pparg2*, *Gata3*, and *Cebpb* were measured by real-time PCR during 3T3-L1 differentiation. Their expression was normalized to that of the *Rplp0* (*36B4*) control. Values are expressed as mean±S.E.M., *n*=3 per group, *P<0.05

We next determined whether CREB3L4 affects adipogenesis *in vitro*. To investigate the function of CREB3L4, we transfected the nuclear form of *Creb3l4* (*Creb3l4*-N)^13^ into 3T3-L1 cells; these cells showed reduced oil-red O staining (Figure 2a). Constitutive expression of *Creb3l4*-N in 3T3-L1 preadipocytes resulted in a decrease in C/EBP*β* from D0, with a concomitant decrease in C/EBP*α* and PPAR*γ*2 expression (Figure 2b; Supplementary Figure S2). Overexpression of *Creb3l4* decreased mRNA levels of *Cebpa* and *Pparg2*, but that of *Cebpb* did not change in the early stages (Figure 2c; discussed below), suggesting that CREB3L4 has a negative role during DMI-induced differentiation of preadipocytes into adipocyte.

### Suppression of *Creb3l4* expression resulted in accelerated adipocyte differentiation by upregulating PPAR*γ*2 expression in 3T3-L1 preadipocytes

**Figure 3:**
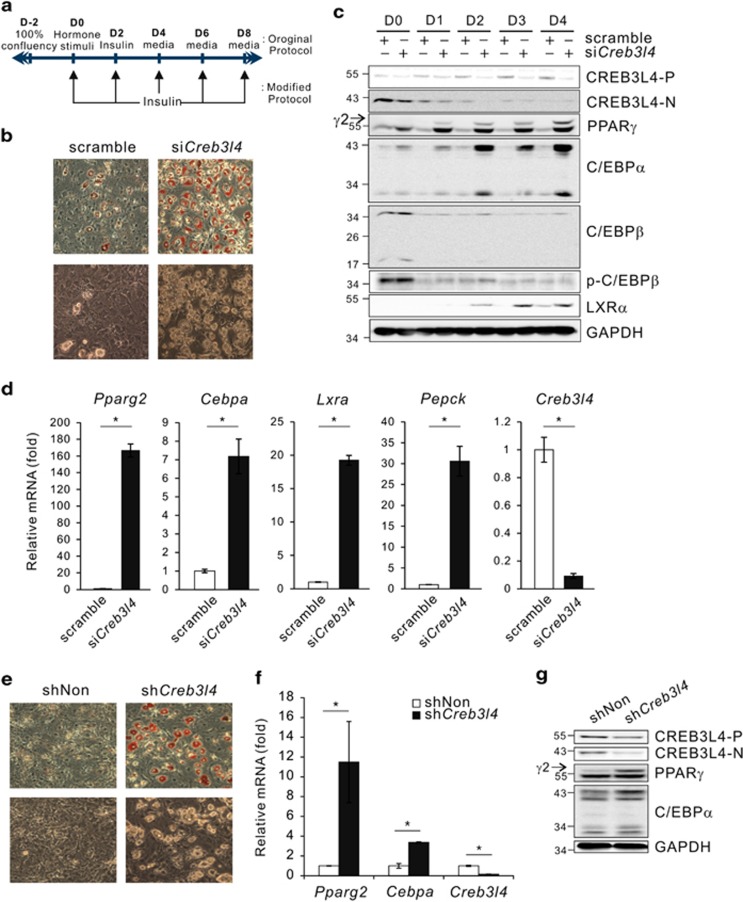
Effect of *Creb3l4* ablation on adipocyte differentiation. (**a**) Modified protocol for inducing adipocyte differentiation. Induction of adipogenesis using the modified protocol in si*Creb3l4*-expressing 3T3-L1 cells treated with insulin (1 *μ*g/ml)-containing media (10% FBS-DMEM) every 2 days. (**b**) After 8 days, the cells were stained with oil-red O, and microscopy ( × 20) was performed. Microscopic views representative of three independent experiments are shown. (**c**) Immunoblot of adipogenic transcription factors at the indicated time points after induction of 3T3-L1 differentiation by insulin. (**d**) The mRNA levels related to lipogenesis, *lxra* and *Pepck*, and adipogenesis, *Pparg2* and *Cebpa*, were determined by real-time PCR at D6. (**e**) Oil-red O staining of stable 3T3-L1 cells expressing sh*Creb3l4* at D8, and microscopy ( × 20) was performed. The 3T3-L1 cells were transduced with lentiviral sh*Creb3l4* and treated with 10% FBS-DMEM media containing insulin (1 *μ*g/ml) every 2 days. Real-time PCR (**f**) and immunoblot (**g**) of adipogenic gene expression in 3T3-L1 cells expressing sh*Creb3l4* at D0. Their expression was normalized to that of the *Rplp0* (*36B4)* control. GAPDH was used as a loading control. Data are representative of three independent experiments. Values are expressed as mean±S.E.M., *n*=3 per group, *P<0.05

If CREB3L4 has a negative role in adipogenesis in preadipocytes, then inhibition of *Creb3l4* expression should result in accelerated differentiation into adipocytes. To test this hypothesis, we examined the adipocyte differentiation capacity of cells under the influence of a minimal hormonal inducer, which suppressed differentiation in the scramble group (Figure 3a; Supplementary Figure S3a). To this end, 3T3-L1 cells that had been transfected with scramble small-interfering RNA (siRNA) or *Creb3l4*-siRNA (si*Creb3l4*) were mock treated, or treated with dexamethasone or insulin in Dulbecco's modified Eagle's medium (DMEM) containing fetal bovine serum (10% FBS-DMEM). The scramble group did not differentiate into adipocytes in the presence of the minimal inducer of differentiation, with mock, insulin, or dexamethasone treatment, whereas in the absence of the inducer, the si*Creb3l4* group exposed to mock treatment differentiated partially (Supplementary Figure S3b). Furthermore, modified differentiation methods were sufficient to induce adipocyte differentiation in the si*Creb3l4* group (Figure 3b; Supplementary Figure S3b). si*Creb3l4*-transfected cells that had been cultured in the presence of insulin showed an increase in protein levels of adipogenic transcription factor (Figure 3c), and PPAR*γ*2 and C/EBP*α* levels were already elevated on D0. The mRNA level of *Pparg2*, *Cebpa*, and lipogenic genes for adipocyte differentiation, *lxra* and *Pepck*, were also induced in si*Creb3l4*-transfected 3T3-L1 cells (Figure 3d).

To further confirm the role of CREB3L4 in adipogenesis, we established a stable cell line expressing *Creb3l4-*shRNA (sh*Creb3l4*); these cells showed increased oil-red O staining when cultured in the presence of insulin (Figure 3e). In addition, sh*Creb3l4* cells showed increased expression of PPAR*γ*2 and C/EBP*α* at D0 (Figures 3f and g). These data indicated that suppression of *Creb3l4* could enhance the capacity of preadipocytes to differentiate into mature adipocytes, and therefore CREB3L4 seemed to be a critical negative regulator, determining preadipocyte–adipocyte transition in 3T3-L1 cells.

### Knockdown of *Creb3l4* results in bypass of the MCE step

**Figure 4:**
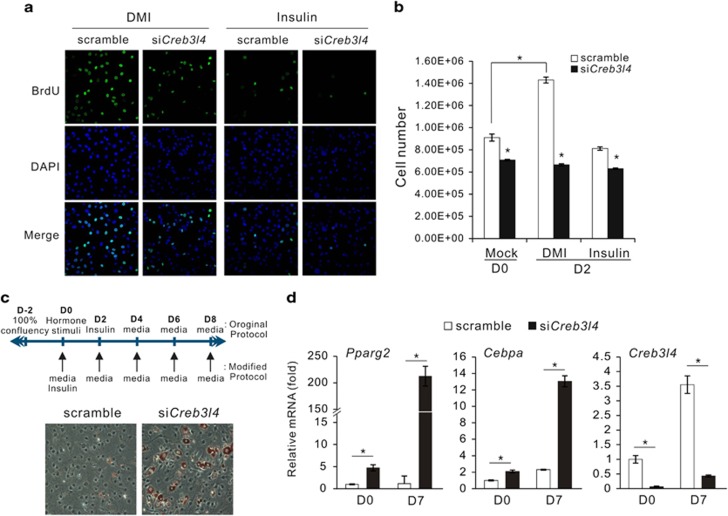
3T3-L1 cells transfected with si*Creb3l4* bypass the mitotic clonal expansion step. (**a**) Immunofluorescence analysis of BrdU-labeled 3T3-L1 adipocytes. The cells were treated with DMI or insulin and were then subjected to BrdU pulsing for 2 h (from 16 to 18 h after initiation of differentiation). The period of the BrdU pulse corresponds to the S phase in induced cells. Representative images of fluorescence microscopy ( × 40 magnification). (**b**) Cell counts were determined at days 0 and 2 after induction of differentiation with 10% FBS-DMEM containing DMI or insulin (1 *μ*g/ml) after transfection of si*Creb3l4*. (**c**) Modified protocol for deletion of mitotic clonal expansion step. Induction of adipogenesis using the modified protocol in si*Creb3l4*-expressing 3T3-L1 cells treated with insulin (1 *μ*g/ml)-containing media (10% FBS-DMEM) for 2 days, and changed media every 2 days. After 8 days, the cells were stained with oil-red O, and microscopy ( × 20) was performed. (**d**) The mRNA levels of *Pparg2*, *Cebpa*, and *Creb3l4* are determined by real-time PCR. Error bars represent mean±S.E.M., *n*=3 per group, *P<0.05

Since MCE of growth-arrested preadipocytes appears to be necessary for optimal differentiation,^14^ we wondered whether increased differentiation in si*Creb3l4* group with insulin treatment underwent the MCE stage or not. To evaluate whether the si*Creb3l4* group re-entered the cell cycle due to insulin treatment, we performed a Bromodeoxyuridine (BrdU) incorporation assay. DMI treatment of the scramble group resulted in active incorporation of BrdU, whereas treatment with insulin alone or with si*Creb3l4* decreased BrdU incorporation as well as the number of cells (Figures 4a and b). Although the combination of si*Creb3l4* and insulin treatment was not able to increase cell proliferation and cell number, adipocyte differentiation still occurred (Figures 3b, 4a, and b). Treatment of DMI to induce differentiation of 3T3-L1 preadipocytes undergo two sequential rounds of mitosis over the next 2 days.^15^ As illustrated in Figure 4c, when the MCE step was skipped, scramble group did not induce adipogenesis. However, adipogenesis in *Creb3l4* knockdown cells was intact and adipogenic transcription factor, *Pparg2* and *Cebpa*, was increased compared with scramble group (Figures 4c and d). Surprisingly, knockdown of *Creb3l4* in 3T3-L1 cells resulted in bypassing MCE during adipocyte differentiation.

### CREB3L4 increases the expression of *Gata3* and regulates protein stability of C/EBP*β*

**Figure 5:**
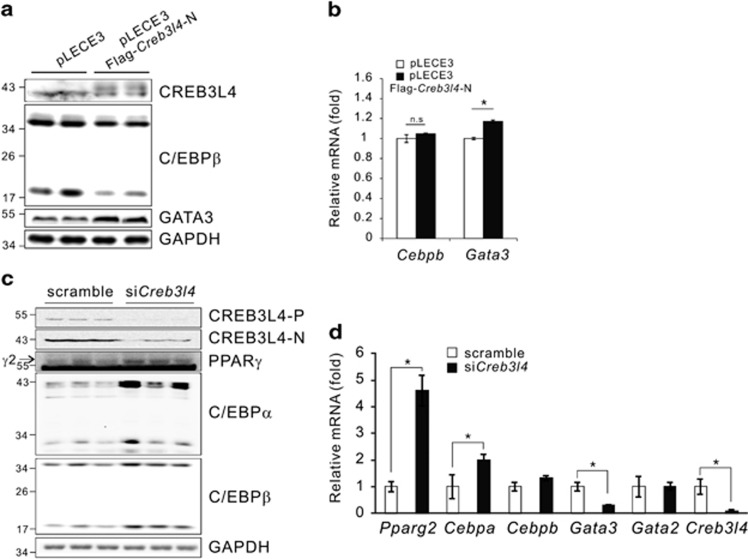
CREB3L4 regulates the stability of C/EBP*β* protein and *Gata3* expression. (**a**, **b**) Immunoblot and real-time PCR analysis of C/EBP*β* and GATA3 expression in 3T3-L1 cells with lentivirus-mediated *Creb3l4 *expression at D0. (**c**) Immunoblot of adipogenic factors at D0 in 3T3-L1 cells transfected with si*Creb3l4*. (**d**) The mRNA levels of transcription factors at D0 in 3T3-L1 cells transfected with si*Creb3l4*. Values are expressed as mean±S.E.M., *n*=3 per group, *P<0.05

As shown in Figures 3c, f, and g, PPAR*γ*2 and C/EBP*α* levels were already elevated on D0. Thus, we assumed that elevated expression of PPAR*γ* and C/EBP*α* in 3T3-L1 preadipocytes causes differentiation into mature adipocytes. Then, we questioned why PPAR*γ*2 and C/EBP*α* were already increased at this time point. We examined the possible mechanisms of action of CREB3L4, including (1) a direct effect of CREB3L4 on the expression of *Pparg2*, *Cebpa*, and *Cebpb* as a repressor; (2) induction of expression of negative regulators by CREB3L4, which in turn would repress *Pparg2* and *Cebpa* expression; and (3) regulation of C/EBP*β* stability, which would affect *Pparg2* and *Cebpa* expression (Figures 2b and c). To answer this question, we measured the effect of CREB3L4 on the promoter activities of *Pparg2*, *Cebpa*, and *Cebpb*, which turned out to have no effect (Supplementary Figure S4a). Furthermore, the mRNA levels of most negative regulators^7^ in the *Creb3l4*-overexpressing group were not altered (Supplementary Figure S4b). However, the expression of *Gata3*, an important negative regulator in preadipocyte–adipocyte transition,^16^ was induced in the *Creb3l4*-transduced cells (Figures 2b, c, 5a, and b). Finally, the protein, but not the mRNA, levels of C/EBP*β* decreased in *Creb3l4*-expressing 3T3-L1 cells at D0 (Figures 2b, 2c, 5a, and 5b). In si*Creb3l4*-treated cells, the protein levels of PPAR*γ*2, C/EBP*α*, and C/EBP*β* also increased at D0 (Figure 5c). However, real-time PCR showed no difference in the *Cebpb* mRNA levels (Figure 5d). These data suggested that CREB3L4 may act as a negative regulator of the differentiation of 3T3-L1 preadipocytes both by upregulating *Gata3* expression and by decreasing the stability of C/EBP*β* protein at D0.

### CREB3L4 induces the C/EBP*β* ubiquitination through protein–protein interaction

**Figure 6:**
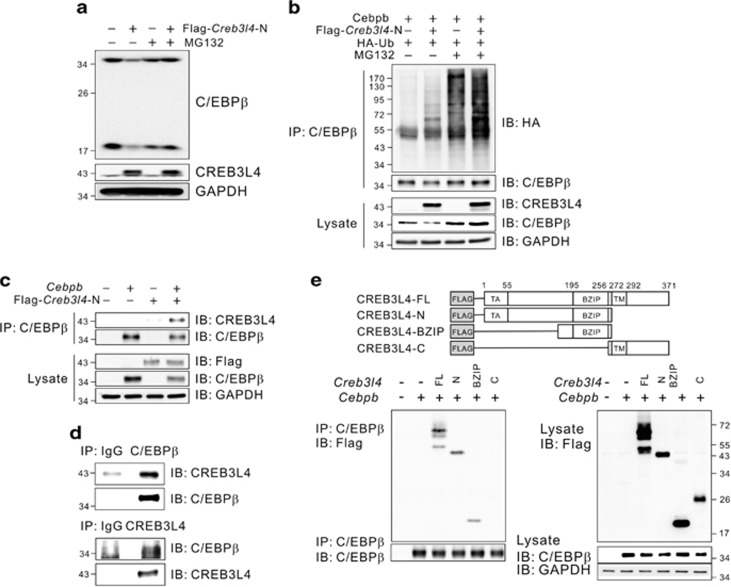
CREB3L4 induces the C/EBP*β* ubiquitination through direct interaction. (**a**) 3T3-L1 cells were transfected with a *Creb3l4*-N expression vector and treated with MG132 (10 *μ*M) for 4 h. C/EBP*β* expression at day 0 (D0) was detected by immunoblotting. (**b**) NIH-3T3 cells were co-transfected with *Creb3l4*, *Cebpb*, and *ubiquitin* expression vectors and then treated for 4 h with MG132 (10 *μ*M). Subsequently, an immunoprecipitation assay was carried out. Ubiquitin-conjugated C/EBP*β* was detected by immunoblotting. (**c**) NIH-3T3 cells transfected with *Cebpb* and *Creb3l4* expression vectors were immunoprecipitated using an anti-C/EBP*β* antibody, or (**d**) their reciprocal antibodies. (**e**) Mapping of the CREB3L4 domain interacting with C/EBP*β*. Each domain of CREB3L4 is shown in the upper panel. NIH-3T3 cells were transfected with *Creb3l4*-FL, *Creb3l4*-N, *Creb3l4*-BZIP, and *Creb3l4*-C with *Cebpb*. After incubation for 48 h, cell lysates were immunoprecipitated with anti-C/EBP*β* antibody and probed with anti-Flag antibody

To examine the effect of CREB3L4 on the stability of C/EBP*β*, we treated *Creb3l4*-transfected 3T3-L1 cells with the proteasomal inhibitor, MG132. The *Creb3l4*-mediated decrease in C/EBP*β* levels was restored in cells treated with MG132 (Figure 6a). Moreover, CREB3L4 induced the ubiquitination of C/EBP*β* (Figure 6b). Because both CREB3L4 and C/EBP*β* have a bZIP domain, we performed an immunoprecipitation assay to investigate their interaction. As expected, CREB3L4 interacted with C/EBP*β* (Figure 6c; Supplementary Figure S5a), and their interaction was confirmed using reciprocal antibodies (Figure 6d). Endogenous C/EBP*β* also interacted with CREB3L4 in 3T3-L1 cells (Supplementary Figure S5b). Subcellular fractionation of cells overexpressing the nuclear form of CREB3L4^13^ and C/EBP*β* revealed that this protein complex is colocalized in the nucleus (data not shown). Interaction between CREB3L4 and C/EBP*β* occurs through their bZIP domains (Figure 6e), suggesting that CREB3L4 induced ubiquitination of C/EBP*β* at D0 through protein–protein interaction.

### C/EBP*β* mediates upregulation of PPAR*γ*2 by inhibition of *Creb3l4*

**Figure 7:**
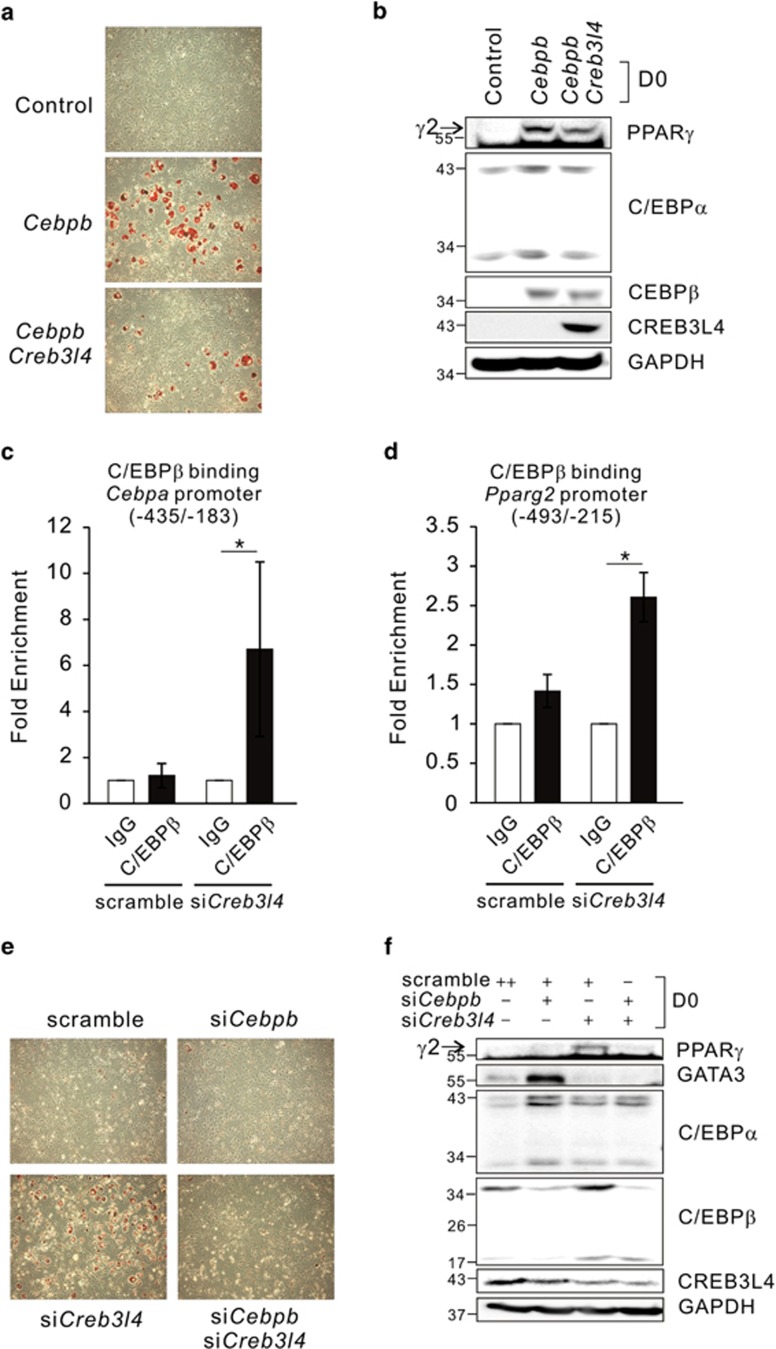
C/EBP*β* is a mediator of CREB3L4 downregulating the expression of PPAR*γ*2. (**a**) Oil-red O staining of adipocytes was performed at D8, and microscopy ( × 20) was performed. 3T3-L1 cells were transfected with *Cebpb* or *Cebpb*/*Creb3l4* and stimulated to differentiate with insulin (1 *μ*g/ml)-containing media (10% FBS-DMEM) every 2 days. (**b**) Immunoblot of adipogenic transcription factors in 3T3-L1 cells expressing *Cebpb* or *Cebpb*/*Creb3l4* at D0. ChIP assay performed in 3T3-L1 cells that transfected with si*Creb3l4* at D0. Normal IgG was used as a negative control for immunoprecipitation. The regions amplified were −435 to −183 bp for *Cebpa* gene promoter (**c**) and −493 to −215 for *Pparg2* gene promoter (**d**). The quantity of immunoprecipitated DNA was normalized to total input DNA. Values are expressed as mean±S.E.M., *n*=3 per group, *P<0.05. (**e**) Oil-red O staining of 3T3-L1 cells transfected with si*Cebpb* and si*Creb3l4* at D8, and microscopy ( × 20) was performed. The cells were stimulated to differentiate as described in (**a**). (**f**) Immunoblot of adipogenic factors in 3T3-L1 cells expressing si*Cebpb* and si*Creb3l4*, at D0

We next determined whether C/EBP*β* could regulate expression of *Pparg2* and *Cebpa* at D0. 3T3-L1 cells transfected with *Cebpb* or *Cebpb*/*Creb3l4* were treated with insulin-containing media every 2 days. Ce*bpb*-transfected cells were differentiated, which was inhibited by co-transfection with *Creb3l4* (Figure 7a; Supplementary Figure S6a). Expression of PPAR*γ*2 and C/EBP*α* was increased by transfection with *Cebpb*, but their expression was suppressed by co-transfection with *Creb3l4* at D0 (Figure 7b; Supplementary Figure S6b). These data are supported by previous reports demonstrating that ectopic expression of *Cebpb* in NIH-3T3 cell induced *Pparg* expression with accompanying adipogenesis.^17^ At D0, stabilized C/EBP*β* in si*Creb3l4* group directly bound to the C/EBP binding region of *Cebpa* and *Pparg2* gene promoter (Figures 7c and d).

To investigate the interrelationship between C/EBP*β* and CREB3L4 in the induction of PPAR*γ*2 and C/EBP*α*, we transfected 3T3-L1 cells with si*Cebpb* and si*Creb3l4*. The cells that had been transfected with si*Creb3l4* showed increased adipocyte differentiation, which was inhibited by si*Cebpb* (Figure 7e; Supplementary Figure S6c). In preadipocytes, si*Creb3l4*-mediated induction of PPAR*γ*2 was decreased by *Cebpb *knockdown (Figure 7f), suggesting that C/EBP*β* is a mediator of si*Creb3l4*-induced PPAR*γ*2 expression in preadipocytes. Because a si*Cebpb*-mediated decrease in PPAR*γ*2 protein levels was difficult to observe at D0, we performed quantitative real-time PCR to investigate *Pparg2* mRNA levels after treatment with si*Cebpb*. *Cebpb *knockdown resulted in decreased *Pparg2* expression, even at D0 (Supplementary Figure S6d). The si*Creb3l4*-mediated increase in *Pparg2* mRNA levels was also decreased by si*Cebpb* (Supplementary Figure S6d). The expression pattern of *Cidec*, a PPAR*γ*-target gene, was similar to that of *Pparg2* (Supplementary Figure S6d).

### GATA3 also mediates suppression of *Creb3l4*-induced PPAR*γ*2 regulation

**Figure 8:**
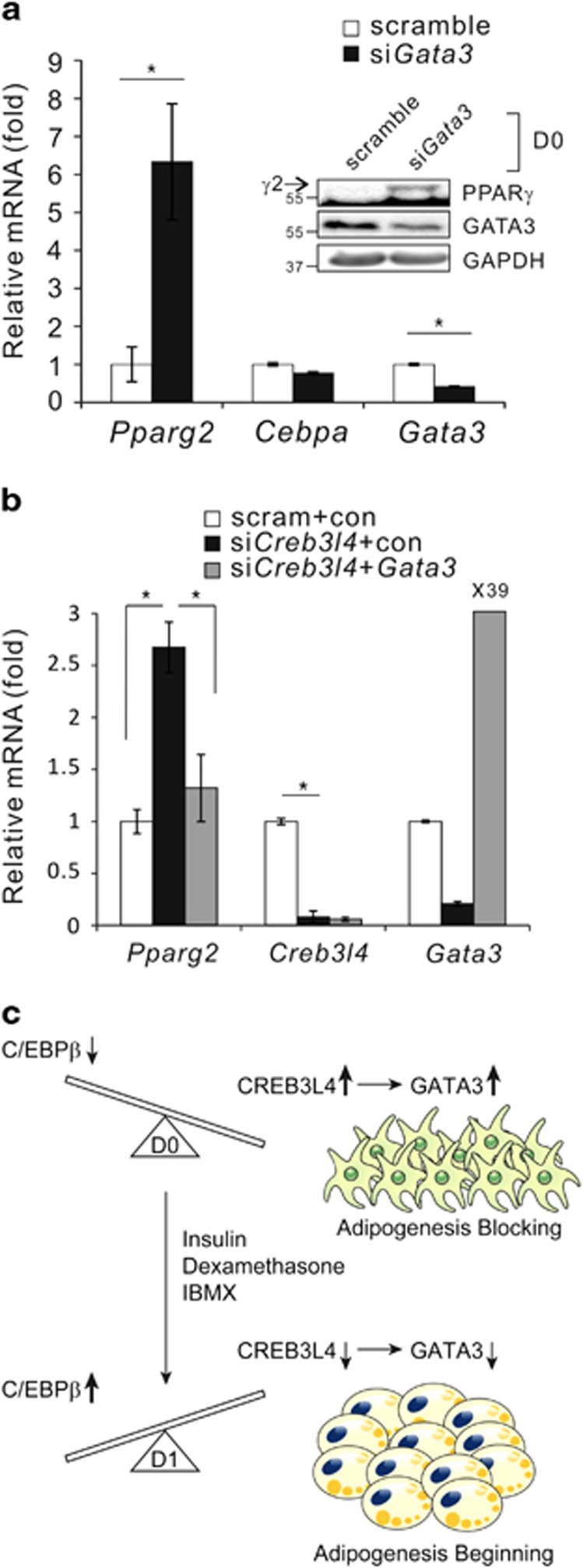
GATA3 is another mediator of CREB3L4 downregulating the expression of PPAR*γ*2. (**a**) Expression of PPAR*γ*2 and C/EBP*α* in 3T3-L1 cells expressing si*Gata3*, at D0. (**b**) The mRNA levels of *Pparg2*, *Creb3l4*, and *Gata3* in 3T3-L1 cells transfected with si*Creb3l4* and a CMV-m*Gata3*-FL expression vector, at D0. Values are expressed as mean±S.E.M., *n*=3, *P<0.05. (**c**) In preadipocytes, CREB3L4 acts as a negative regulator of adipogenesis by both regulating the stability of C/EBP*β* protein and increasing *GATA3* expression. After induction of differentiation by DMI (DMI: 0.5 mM 3-isobutyl-1-methylzanthine [IBMX; M], 1 *μ*M dexamethasone [D], and 1 *μ*g/ml of insulin [I]), *Creb3l4* and *Gata3* expression is decreased, and increased C/EBP*β* levels initiate adipogenesis by increasing the expression of PPAR*γ*2 and C/EBP*α*. Suppression of *Creb3l4* in preadipocytes induces adipogenesis by increasing PPAR*γ*2 expression

Furthermore, because GATA3 is known to be a negative regulator of *Pparg2* expression,^16^ it is possible that downregulation of GATA3 expression by si*Creb3l4* (Figure 5d) could also contribute to the upregulation of PPAR*γ*2 at D0. To test this hypothesis, we transfected 3T3-L1 cells with si*Gata3*; we observed increased expression of PPAR*γ*2, but not that of C/EBP*α* (Figure 8a). In addition, the si*Creb3l4*-mediated increase in *Pparg2* expression was diminished by overexpression of *Gata3* (Figure 8b). These data suggested that GATA3 is another mediator upregulating *Pparg2* expression when *Creb3l4* expression is knocked down. Taken together, these results indicated that CREB3L4 acts as a negative regulator that blocks adipogenesis at D0, by inducing both ubiquitination of C/EBP*β* and expression of *Gata3*.

### *Creb3l4*-KO mice showed improved metabolic parameters due to increased adipogenesis

**Figure 9:**
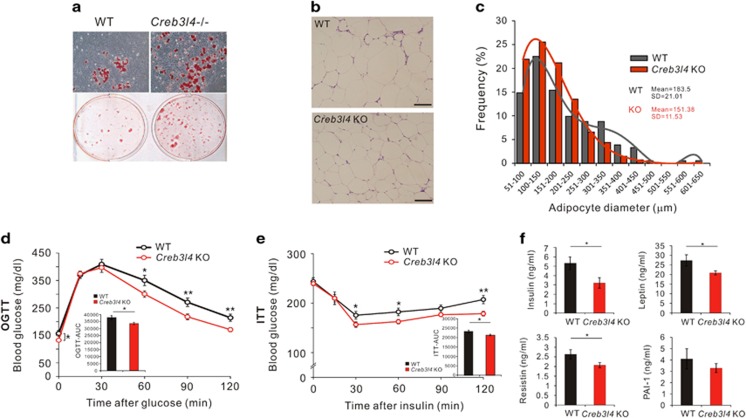
*Creb3l4*-KO mice showed adipocyte hyperplasia, lead to improved metabolic parameters. (**a**) Adipogenic potential of mouse embryonic fibroblasts (MEFs) derived from wild-type (WT) or *Creb3l4*-knockout (KO) mice. Cells were stained with oil-red O after treatment with insulin (10 *μ*g/ml) and rosiglitazone (2 *μ*M) for 6 days, followed by insulin (1 *μ*g/ml) for 16 days. Microscopic ( × 20) views representative of three independent experiments are shown. (**b**) Hematoxylin and eosin (H&E) staining of epididymal fat. Scale bar: 100 *μ*m. (**c**) Adipocyte diameter was quantified with Image J (*n*=3). (**d**) Oral glucose tolerance test (GTT), (**e**) insulin tolerance test (ITT), performed after 14 and 15 weeks, respectively, of feeding HFD (*n*=12–19). (**f**) Insulin level from mice was measured by ELISA ( *n*=9–14 ). Leptin, resistin, and PAI-1 levels from mice fed HFD for 16 weeks were measured by MAGPIX, using a MILLIPLEX MAP mouse magnetic bead panel (*n*=6–12). Values are expressed as mean±S.E.M., *P<0.05, **P<0.01

To further confirm the effects of CREB3L4 in adipogenesis, adipocyte differentiation in *Creb3l4* KO and WT mouse embryonic fibroblasts (MEFs) was compared. Primary *Creb3l4* KO MEFs showed an increase in lipid accumulation and morphological differentiation compared with WT MEFs at D22, even in the insulin-containing medium (Figure 9a). To elucidate the physiological role of *Creb3l4* in adipogenesis, both WT and *Creb3l4-*KO mice were fed with a 45% high-fat diet (HFD) for up to 16 weeks. There are no significant differences in body weight and fat mass (data not shown). However, the size of adipocyte fat cells in *Creb3l4-*KO mice was smaller than that in WT mice (Figures 9b and c). The average of epididymal adipocyte diameter in WT mice was 184 *μ*m, in the *Creb3l4-*KO mice, average of adipocyte size was smaller (151 *μ*m) (Figure 9c). In addition, *Creb3l4-*KO mice exhibited an increased number of these small adipocytes (hyperplasia), which may contribute to improved glucose tolerance and insulin sensitivity (Figures 9d and e). HFD-fed *Creb3l4-*KO mice showed decreased fasting glucose levels (Figure 9d) and were resistant to diet-induced hyperinsulinemia (Figure 9f). However, plasma lipid profiles, including triglyceride, total cholesterol, and non-esterified free fatty acid levels, were not significantly different between these groups (Supplementary Figure S7a). Because adipose tissue can affect systemic glucose homeostasis by secreting adipokines, we measured adipokine levels. We found lower levels of leptin, resistin, and PAI-1 in *Creb3l4-*KO mice than in WT mice, suggesting that deletion of *Creb3l4* led to an altered adipokine profile that may contribute to improved insulin sensitivity (Figure 9f). In addition, insulin-stimulated Akt phosphorylation (S473) was augmented in WAT and liver tissues, but not in muscle, of the *Creb3l4-*KO mice (Supplementary Figure S7b). These results indicate that adipocyte hyperplasia due to increased adipogenesis in HFD-fed *Creb3l4-*KO mice could be an adaptation to overcome glucose intolerance and insulin resistance.

## Discussion

Adipose tissue serves as an important regulator of metabolic homeostasis, and thus, accumulation of fat depots is associated with insulin resistance and a high risk for type 2 diabetes and metabolic syndrome.^18^ Studying the biological networks and transcription factors, especially negative regulators, that control adipogenesis is important in the development of a therapeutic strategy for obesity-associated type 2 diabetes and metabolic syndrome. In the present study, we explored the role of CREB3L4 in adipogenesis and defined a novel mechanism regulating initiation of adipogenesis. Suppression of *Creb3l4* resulted in the expression of PPAR*γ*2 and C/EBP*α*, followed by initiation of adipogenesis even in the presence of minimal hormonal inducer in 3T3-L1 preadipocytes, and induced hyperplastic WAT with the improved metabolic parameters.

Although there was a difference in the degree of adipocyte differentiation depending on the stimuli used, viz., insulin or dexamethasone, adipogenesis did occur in all of the si*Creb3l4*-treated groups (Figure 3b; Supplementary Figure S3b). Because insulin is known to be a strong inducer of lipogenic gene expression, insulin treatment of si*Creb3l4*- and sh*Creb3l4*-transfected cells resulted in accelerated adipogenesis, as compared with the mock- or dexamethasone-treated group. Insulin strongly induces the expression of *Add1/Srebp1*, which, in turn, promotes adipocyte differentiation by augmenting PPAR*γ*-mediated gene expression.^19^ Indeed, ADD1/SREBP1 and PPAR*γ* were shown to cooperate to produce PPAR*γ* ligand and to stimulate *Pparg* expression.^20, 21^ These data suggested that CREB3L4 could be a critical negative regulator determining preadipocyte–adipocyte transition in 3T3-L1 cells and that knockdown of this gene could promote adipogenesis.

At D0, it seemed that elevated expression of PPAR*γ* and C/EBP*α*, by suppression of *Creb3l4*, resulted in transition of 3T3-L1 preadipocytes to mature adipocyte. These data are supported by previous reports that demonstrated that retroviral expression of *Pparg2* induced differentiation of cultured fibroblasts into adipocytes, by cooperating with C/EBP*α* in stimulating the adipocyte program. Moreover, PPAR*γ* alone can initiate the entire adipogenesis program in adipocytes.^7, 22^ Thus, it is likely that elevation of PPAR*γ*2 and C/EBP*α* due to *Creb3l4*-knockdown could initiate adipogenesis by sequentially inducing expression of genes that are involved in adipocyte differentiation, even in the presence of minimal hormonal inducer.

The increase in PPAR*γ*2 and C/EBP*α* expression may have further significance. Inhibition of *Creb3l4* in 3T3-L1 preadipocytes decreased BrdU incorporation and resulted in a lack of further increase in cell number after treatment with DMI or insulin alone, which seemed to involve increased expression of C/EBP*α* and PPAR*γ*2. C/EBP*α* has anti-mitotic and anti-proliferative actions in terminally differentiating adipocytes and myeloid cells.^23, 24^ Ectopic and endogenous expression of *Pparg* is also sufficient to cause cell-cycle arrest, by modulating E2F phosphorylation.^25^ Additionally, PPAR*γ* and C/EBP*α* can promote growth arrest by upregulating the Cdk inhibitor.^26, 27^ Thus, PPAR*γ*2 and C/EBP*α* expression by si*Creb3l4* caused adipocyte differentiation, bypassing the MCE step for adipogenesis.

Our study collectively demonstrates that CREB3L4 tightly controls the preadipocyte to adipocyte transition through a dual mechanism, viz., decreasing protein stability of C/EBP*β* through ubiquitination, and upregulation of *Gata3* expression. It was unclear why CREB3L4 induces ubiquitination of C/EBP*β* at D0. Presumably, because C/EBP*β*, as a transcription factor, induces expression of downstream adipogenic transcription factors, CREB3L4 may induce C/EBP*β* ubiquitination to maintain low levels of C/EBP*β* to retain preadipocyte status. CREB3L4 could induce the ubiquitination of C/EBP*β*, but further study, which E3 ligase is involved with CREB3L4, is required to understand the ubiquitin conjugation mechanism for C/EBP*β*, including ubiquitin ligase. In addition, we observed that knockdown of C/EBP*β* expression also decreased the expression of CREB3L4 (Figure 7f; Supplementary Figure S6d). These data suggest that C/EBP*β* and CREB3L4 are co-regulated with each other. Thus, there may exist positive or negative transcription factors regulating the expression of C/EBP*β* and CREB3L4, respectively. Because the regulation of CREB3L4 during differentiation is not known at present, molecular mechanism(s) of how C/EBP*β* downregulates CREB3L4 need to be explored. Further studies on the interplay between C/EBP*β* and CREB3L4 may help understand their role in the adipocyte differentiation. In addition, GATA3 expression is decreased by treatment of si*Creb3l4* in 3T3-L1 preadipocytes, resulting in upregulation of PPAR*γ*2, but not of C/EBP*α* (Figure 8a), which indicates that GATA3 affects only *Pparg2* expression. These data are consistent with a previous report that GATA3 acts as a negative regulator of adipocyte differentiation through inhibition of *Pparg* expression, by directly binding to the *Pparg2* promoter.^16^ *Gata3*-deficient ES cells also showed enhanced capacity of adipocyte differentiation under minimally permissive hormonal condition.^16^ Our study suggests that CREB3L4 could be upstream factor for *Gata3* gene expression in preadipocytes. In addition, GATA3 is capable of forming protein complexes with either C/EBP*α* or C/EBP*β*, resulting in the suppression of adipocyte differentiation.^28^ Because GATA3, C/EBP*α*/*β*, and CREB3L4 are closely associated with the initiation of adipogenesis, the interrelationship among these molecules needs to be identified in detail to understand the initiation of adipogenesis.

In general, adipose tissue expands by increased adipocyte size (hypertrophy) and number (hyperplasia).^29^ However, it is known that HFD-induced adipose tissue expansion is triggered mainly by hypertrophy during the first month of HFD feeding. After prolonged HFD exposure (i.e., longer than 1 month), Epi-WAT showed a high adipogenesis rate as determined by a large number of new fat cells (hyperplasia).^30^ Differentiated adipocytes are post-mitotic; therefore, hyperplasia represents an increase in *de novo* adipocyte formation (adipogenesis). *Creb3l4-*KO mice showed increased adipogenesis compared with WT littermates (Figures 9b and c), similar to the findings observed in 3T3-L1 cells. Consequentially, HFD-fed *Creb3l4-*KO mice exhibited improved metabolic parameters due to the increase in adipocyte number (Figures 9d–f).

However, the phenotype of the *Creb3l4* KO mice is mild. Because *Creb3l4* is expressed in other tissues, the generation and characterization of adipocytes-specific knockout mice will be required to determine the roles of CREB3L4 in obesity and related metabolic diseases.

Taken together, we demonstrated a role for CREB3L4 in the differentiation of 3T3-L1 preadipocytes, which functions as a negative regulator, blocking adipogenesis. In these preadipocytes, CREB3L4 increased the expression of *Gata3* as well as the ubiquitination of C/EBP*β*, to retain the preadipocyte status of the cells. When the cells were exposed to adipogenic inducers (DMI), *Creb3l4* expression was decreased, leading to commencement of adipogenesis. It is likely that *Creb3l4* expression determines the fate of cells to differentiate into adipocytes (Figure 8c). Furthermore, CREB3L4 and C/EBP*β* genes express contrariwise during the differentiation process^31^ and to the component of the differentiation cocktail (Figure 1d). Turning off of CREB3L4 expression is a prerequisite to turning on the C/EBP*β* expression. Thus, it is speculated that a balance between these proteins is critical for adipocyte differentiation (Figure 8c). Indeed, *Creb3l4-*KO mice subjected to an HFD showed increased hyperplastic WAT, due to enhanced adipogenesis, which may contribute to overcome HFD-induced glucose intolerance and insulin resistance. These results raise the possibility that *Creb3l4* could be a useful therapeutic target in the fight against obesity and metabolic syndrome.

## Materials and Methods

### Animal experiments and KO mice

SVF and AdipoF were isolated from epididymal fat pads of 12-week-old C57BL/6J (C57BL/6JJmsSlc) mice. Briefly, the fat pads were rapidly excised, finely minced, and incubated at 37 °C for 1 h with 1 mg/ml type I collagenase in Hank's buffered salt solution containing 1% BSA, 200 nM adenosine, and 50 mg/ml glucose. After sequential filtration and centrifugation, floating adipocytes (AdipoF) and pellet (SVF) were separated.^32^ *Creb3l4*-KO mouse embryos were purchased from RIKEN (The Institute of Physical and Chemical Research, Ibaraki, Japan) with permission from Dr Nojima. The *Creb3l4-*KO mice have been described previously.^10^ At 11 weeks of age, *Creb3l4*-KO mice and their WT littermates were fed a 45% HFD (Research Diets, New Brunswick, NJ, USA) for 18 weeks. To test activation of insulin signaling pathway, mice fed HFD were fasted for 6 h and then received insulin (5 U/kg) by intraperitoneal injection. After 5 min, the mice were killed.^33, 34^ All the animal experiments were approved by the Institutional Animal Care and Use Committee of Yonsei University College of Medicine.

### Glucose tolerance and insulin tolerance tests

For oral glucose tolerance tests, mice were fasted for 16 h and then challenged with oral gavage of glucose (2 g/kg body weight). For insulin tolerance tests, mice were fasted for 6 h and then given an intraperitoneal injection of insulin (0.75 U/kg body weight; Humulin R, Eli Lilly, Indianapolis, IN, USA). Blood glucose levels were measured, by using a glucose monitor (One TOUCH Sure Step, Life Scan, Milpitas, CA, USA), in blood drawn from the tail vein. The area under the curve of glucose was calculated during the course of the tests.

### Phenotypic evaluation of mice

Plasma insulin levels were then measured using ELISA kits (ALPCO Immunoassays, Salem, NH, USA). Plasma adipokines were measured using MAGPIX (Luminex, Austin, TX, USA) with MILLIPLEX MAP mouse magnetic beads (Merck Millipore Corp., St Charles, MO, USA). Other plasma metabolites, such as cholesterol, triacylglycerol, and NEFA were measured by an enzymatic method using an autoanalyzer (Hitachi 7600; Hitachi Instruments, Tokyo, Japan) as per the manufacturer's instructions.

### Cell culture and induction of differentiation

3T3-L1 preadipocytes were cultured in DMEM containing 10% calf serum (10% CS-DMEM), 100 U/ml penicillin, 100 *μ*g/ml streptomycin, and 8 *μ*g/ml biotin, until they reached confluence, and then the cells were maintained for an additional 2 days in the same medium. These post-confluent cells (day 0 [D0]) were stimulated to differentiate using DMEM containing 10% fetal bovine serum (10% FBS-DMEM), and a mixture of 0.5 mM 3-isobutyl-1-methylxanthine, 1 *μ*M dexamethasone, and 1 *μ*g/ml of insulin (DMI) for 2 days. Cells were then further maintained for 2 days in 10% FBS-DMEM containing only 1 *μ*g/ml of insulin. After D4, the media were replaced every other day with 10% FBS-DMEM without insulin. To suppress differentiation in the scramble group, the adipocyte differentiation protocol was modified. Briefly, the cells were incubated with 10% FBS-DMEM containing mock (no treatment), insulin (1 *μ*g/ml), or dexamethasone (1 *μ*M), respectively, from D0, and media were changed every 2 days until D8, while maintaining the respective hormonal stimulation. For MEF studies, heterozygous animals were bred to generate E13.5 embryos. Primary MEFs (passages 1–2) obtained from E13.5 embryos were grown to confluency (D0) in 10% FBS-DMEM. Adipocyte differentiation was induced by treating cells with 10% FBS-DMEM containing insulin (10 *μ*g/ml) and rosiglitazone (2 *μ*M) for 6 days. Thereafter, the cells were maintained in 10% FBS-DMEM containing only insulin (1 *μ*g/ml) for 16 days.

### Total RNA isolation and quantitative real-time PCR

Total RNA was isolated from mouse tissues using TRIzol reagent (Invitrogen, Carlsbad, CA, USA), and total RNA of 3T3-L1 cells was isolated using the Easy Spin RNA extraction kit (iNtRON, Gyeonggi-do, South Korea) according to the manufacturer's instructions. This RNA was used to generate cDNA using the ImProm-II Reverse Transcription System (Promega, Madison, WI, USA). Quantitative real-time PCR (qPCR) was performed using the Step One Real-Time PCR Systems instrumentation and software (Applied Biosystems, Foster City, CA, USA) according to the manufacturer's protocol. The relative amount of mRNA in each sample was normalized to *Rplp0* (*36B4*) transcript levels. The sequences for the gene-specific PCR primers are listed in Supplementary Table S1.

### Western blotting

Proteins from 3T3-L1 and NIH-3T3 cells were isolated using the Pro-Prep protein extraction solution (iNtRON) containing appropriate protease inhibitors. The lysate was heated at 100 °C for 10 min, centrifuged for 5 min at 13 000 r.p.m. to remove the cell debris. Protein concentration was determined using BCA protein assay (Thermo Scientific, Rockford, IL, USA). The protein (50 *μ*g) in each lane was subjected to sodium dodecyl sulfate-polyacrylamide gel electrophoresis (SDS-PAGE) and transferred onto nitrocellulose membranes (Whatman, Dassel, Germany). Membranes were blocked with 5% non-fat milk and incubated with the following primary antibodies: anti-CREB3L4 (AT1618a, Abgent Inc., San Diego, CA, USA), anti-C/EBP*β* (sc-150), anti-C/EBP*α* (sc-61), (Santa Cruz Biotechnology Inc., Santa Cruz, CA, USA), anti-AKT (pan) (#4691s), anti-p-AKT (#9271s), anti-GAPDH (#2118), anti-PPAR*γ* (#2443), anti-p-C/EBP*β* (#3084) (all from Cell Signaling, St Louis, MO, USA), anti-GATA3 (ab106625, Abcam, Cambridge, MA, USA), anti-HA (#11583816001, Roche, Mannheim, Germany), and anti-Flag (F3165, Sigma-Aldrich, St Louis, MO, USA), and anti-LXR*α* ^35^ antibodies. Membranes were then incubated with anti-mouse or anti-rabbit goat horseradish peroxidase-conjugated secondary antibodies (Thermo Scientific) at a 1 : 4000 dilution, in 5% non-fat milk in PBST buffer (137 mM NaCl, 2.7 mM KCl, 10 mM Na_2_HPO_4_, 2 mM KH_2_PO_4_, 0.1% Tween-20), for 1 h at room temperature. Target proteins were visualized using an enhanced chemiluminescence detection system (West Pico & West Dura, Thermo Scientific). The protein bands were detected using a Fujifilm LAS-3000 Imager (FUJIFILM Corporation, Tokyo, Japan).

### Ubiquitination assay

Cells were transfected with Flag-tagged *Creb3l4* and HA-tagged *ubiquitin* using FuGENE HP transfection reagent (Promega Corporation). After 48 h, cells were treated with 10 *μ*M MG132 for 4 h before harvesting, and were then resuspended in cell lysis buffer (50 mM Tris (pH 7.4), 150 mM NaCl, 0.2% Triton X-100, 0.3% NP-40) containing protease-inhibitor cocktail tablets (Roche). The lysates were centrifuged and precleared with protein G-agarose (#05015952001, Roche), followed by a 2-h incubation with anti-C/EBP*β* antibody (Santa Cruz Biotechnology Inc.) and a 1-h incubation with protein G-agarose at 4 °C. Beads were washed twice with washing buffer (1/3 dilution of cell lysis buffer in phosphate-buffered saline (PBS)), and briefly centrifuged and resuspended in the sample buffer before being subjected to SDS-PAGE.

### Immunoprecipitation assay

Constructs expressing Flag-tagged *Creb3l4* and *Cebpb* were co-transfected into NIH-3T3 cells using FuGENE HP transfection reagent. After 48 h, cells were harvested and lysed in cold cell lysis buffer (50 mM Tris (pH 7.4), 150 mM NaCl, 0.2% Triton X-100, 0.3% NP-40) containing appropriate protease inhibitors. Whole-cell lysate (800 *μ*g) was precleared with protein G-agarose (#05015952001, Roche) followed by incubation with anti-C/EBP*β* antibody or anti-CREB3L4 antibody for 16 h and protein G-agarose for 2 h at 4 °C. After centrifugation, the protein G-agarose pellets were washed several times with washing buffer (1/3 dilution of cell lysis buffer in PBS) and resuspended in sample buffer before being subjected to SDS-PAGE.

### Small-interfering RNA

RNA oligonucleotides for scramble (forward, 5′-CCUACGCCACCAAUUUCGUdTdT-3′) (Bioneer), mouse *Creb3l4* (forward, 5′-UUUCUGAGCAGUGUAUCAUAUUGGG-3′), mouse *Gata3* (forward, 5′-CAUGAAGCUGGAGACGUCUCACUCU-3′), and mouse *Cebpb* (forward, 5′-AGUAGAAGUUGGCCACUUCCAUGGG-3′) (Invitrogen) were synthesized. Each siRNA (30 nM) was transfected into appropriate experimental sets of 3T3-L1 cells using Lipofectamine RNA iMAX (Invitrogen) for at least 48 h; cells were then lysed for RNA and protein preparation.

### Construction of plasmids

Expression constructs encoding the full-length (FL), nuclear (N), BZIP (BZIP), and C-terminal (C) forms of mouse *Creb3l4* were PCR amplified and cloned directly into the pcDNA3.1-Flag2 vector. Similarly, the PCR-amplified fragments of *Cebpb* were inserted into the *Bam*HI/*Xho*I sites of an expression vector, pcDNA3.1 mC*ebpb*-V5/His. To construct luciferase reporters, mouse *Cebpb* regions −1951/+181 and mouse *Cebpa* regions −1884/+220 were subcloned into the *Kpn*I/*Xho*I sites of pGL4.14. The mouse *Pparg2* promoter covering the region −2000/+27 was a generous gift from Dr JW Kim.^36^ CMV-mGATA3-FL, encoding the full-length mouse *Gata3* was a gift from Dr GR Lee.^37^ HA-tagged ubiquitin expression vector was a gift from Dr HG Yoon.^38^ A lentiviral expression vector, pLECE3-m*Creb3l4*-N, was constructed by subcloning the mouse nuclear form of *Creb3l4* into the *Hp*aI/*Not*I sites of pLECE3. The lentiviral vector pLECE3 was a kind gift from Dr KH Chun.^39^ The lentiviral vector pLKO.1-shRNA-*Creb3l4* (TRCN0000086183) and pLKO.1 puro Non-Target shRNA were purchased (Sigma-Aldrich). The shRNA sequence of *Creb3l4* was as follows: 5′-CCGG;GCACCTCAAATGCTTGTCATACTCGAGTATGACAAGCATTTGAGGTGCTTTTTG-3′.

### BrdU incorporation assay

BrdU labeling was performed on cells induced to differentiate on coverslips with DMI or insulin alone, using a 5-Bromo-2′-deoxy-uridine Labeling and Detection Kit I (Roche) according to the manufacturer's instructions. The coverslips were subsequently mounted for immunofluorescence analysis.

### Establishment of a stable cell line

The lentivirus particles were generated using three plasmids, VSV (pMDG), RSV-REV, and PMDLg/pRRE, in which 293FT cells were co-transfected with pLECE3-Flag-m*Creb3l4*(N) (control-pLECE3) or pLKO.1-shRNA-*Creb3l4* (control-pLKO.1 puro Non-Target shRNA). The 293FT cells were transfected using Plus-Lipofectamine transfection reagent (Invitrogen) and the media was replaced with 10% CS-DMEM. Two days after transfection, the cell culture media was filtered using a 0.45-*μ*m syringe filter. Culture media for 3T3-L1 cells were changed to a 1 : 5 mixture of viral supernatant and fresh 10% CS-DMEM containing polybrene (H9268, Sigma-Aldrich) at a final concentration of 8 *μ*g/ml. After 3 days, lentivirus-infected 3T3-L1 cells were selected using 2 *μ*g/ml of puromycin (Sigma-Aldrich).

### Chromatin immunoprecipitation assay

Chromatic immunoprecipitation (ChIP) experiments were performed as previously described^35^ with 3T3-L1 cells transfected with scramble or si*Creb3l4* at D0. Briefly, protein extracts were incubated for 15 h at 4 °C with 6 *μ*g anti-C/EBP*β* (sc-150, Santa Cruz Biotechnology Inc.) or normal rabbit IgG (sc-2027, SantaCruz Biotechnology Inc.) antibodies. Protein and/or DNA complexes were precipitated for 1 h at 4 °C using 60 *μ*l of 50% (vol/vol) protein G agarose/salmon sperm DNA slurry. DNA fragments were purified using a PCR purification kit (28106; Qiagen, Valencia, CA, USA) and measured by quantitative real-time PCR. All reactions were normalized relative to input activities to account for chromatin sample preparation differences (ΔC_t_) and determined to the difference between the *α*-C/EBP*β* IP sample (ΔC_t [C/EBP*β*]_) and *α*-IgG IP sample (ΔC_t [IgG]_) for fold enrichment. ΔΔC_t [C/EBP__*β*−__IgG]_=ΔC_t [C/__EBP*β*]_−ΔC_t [IgG],_ Fold change in occupancy=2^(^^−ΔΔCt[C/EBP*β*^^-IgG])^.^40, 41^ The primers used for the PCR of the promoter of C/EBP binding regions of *Pparg2* gene (F-5′-TTCAGATGTGTGATTAGGAG-3′, R-5′-AGACTTGGTACATTACAAGG), and *Cebpa* gene (F-5′-TCCCTAGTGTTGGCTGGAAG-3′, R-5′-CAGTAGGATGGTGCCTGCTG-3′).^42^

### Electroporation of 3T3-L1 preadipocytes and transient transfection

To maximize the transfection efficiency, 3T3-L1 preadipocytes were transfected using an OneDrop Microporator MP-100 (Digital Bio, Seoul, South Korea). The cells were transfected using a pipette-type Neon Transfection System according to the manufacturer's instruction (MPK10096, Invitrogen). The cells were electroporated at 1300 V, using two pulses with a 20-ms pulse width. NIH-3T3 cells were plated in 12-well tissue culture dishes at a density of 2 × 10^5^ cells/well in 1 ml DMEM. Expression plasmids for *Creb3l4* (0, 50, 100, and 200 ng), luciferase-tagged m*Pparg2* −2000/+27, m*Cebpb* −1951/+181, m*Cebpa* −1884/+220 (500 ng), and *Renilla* luciferase were co-transfected using FuGENE HP Transfection Reagent (Promega) at a ratio of 3 : 1. The total amount of transfected plasmid was adjusted to 200 ng by adding empty vector. All luciferase experiments were performed using a Dual-Luciferase Reporter Assay System (Promega). Luciferase activities were normalized to *Renilla* luciferase activities to adjust transfection efficiency.

### Hematoxylin and eosin staining

Epi-WAT was fixed with 10% neutral-buffered formalin, embedded in paraffin, and sectioned. Hematoxylin and eosin (H&E) staining was then performed on these sections.

### Statistical analysis

Three to five experiments were performed for *in vitro* studies, using triplicate replicates of each transfection. The data are represented as means±standard error of the mean (S.E.M.). All data of *in vitro* and *in vivo* sets were analyzed for statistical significance using non-parametric Mann–Whitney tests and independent samples *t*-Test, respectively. All *P-*values<0.05 were considered as significant. Statistical analysis was performed using SPSS (IBM SPSS statistics ver. 20; IBM Corp., Armonk, NY, USA).

## Abbreviations

CREB3L4: cyclic AMP-responsive element-binding protein 3 (CREB3)-like 4
PPAR: peroxisome proliferator-activated receptor
MEF: mouse embryonic fibroblast
C/EBP: CCAAT/enhancer binding protein
MCE: mitotic clonal expansion
