# Supplementary material for: Identification of Creb3l4 as an essential negative regulator of adipogenesis
Source: Cell Death Dis. 2014 Nov 20;5(11):e1527–. doi: 10.1038/cddis.2014.490 (PMC4260748; doi:10.1038/cddis.2014.490)
Supplement: Supplementary Information [file cddis2014490x1.pdf]

Supplementary Information

Identification of *Creb3l4* as an essential negative regulator of adipogenesis

Tae-Hyun Kim[1], Seong-Ho Jo[1, 2], Hyeonjin Choi[1], Joo-Man Park[1], Mi-Young Kim[1], Hiroshi Nojima[3], Jae-Woo Kim[1, 2], Yong-Ho Ahn[1, 2, *]

[1]Department of Biochemistry and Molecular Biology, Yonsei University College of Medicine, Seoul 120-752, Republic of Korea

[2]Brain Korea 21 PLUS Project for Medical Sciences, Yonsei University College of Medicine, Seoul 120-752, Republic of Korea

[3]Department of Molecular Genetics Research Institute for Microbial Diseases Osaka University, Osaka, Japan

*__Corresponding author__: Yong-Ho Ahn, MD, PhD;
Dept. of Biochemistry and Molecular Biology,
Yonsei University College of Medicine,
50-1 Yonsei-ro, Seodaemun-gu, Seoul 120-752, Republic of Korea.
Tel: +82–2–2228–0835, Fax: +82–2–312–5041,
e-mail: yha111@yuhs.ac

Supplementary Figure S1

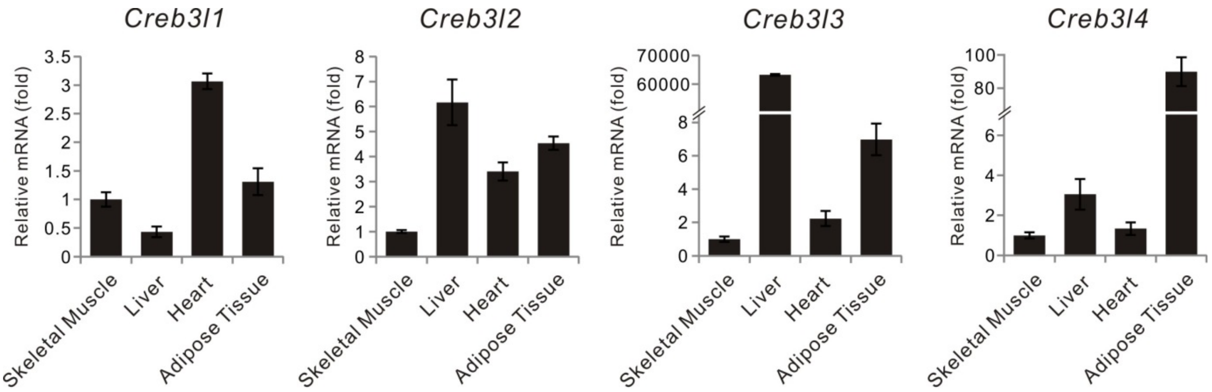

Supplementary Figure S1. Expression of CREB3L-family members in mice. The mRNA levels of *Creb3l* family members in the liver, heart, muscle, and white adipose tissue (WAT) was measured by real-time PCR; expression levels were normalized to that of the *Rplp0* (*36B4)* control. Error bars represent mean ± SD.

Supplementary Figure S2

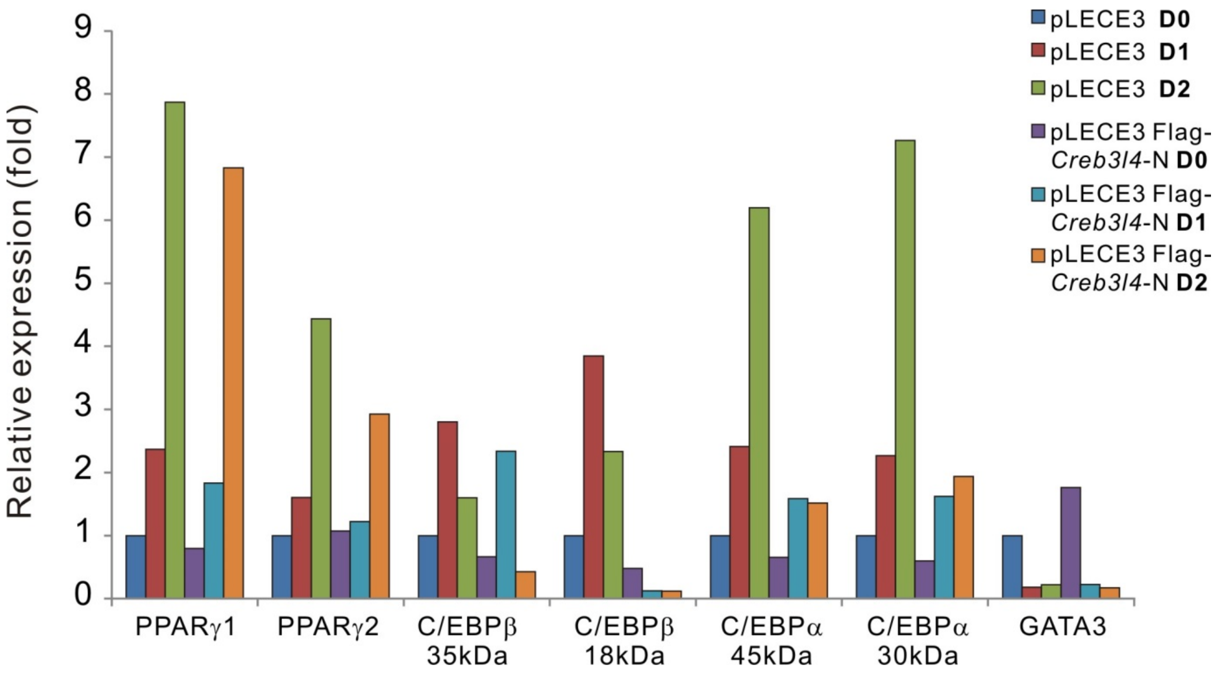

Supplementary Figure S2. Quantification analysis of western blot data from Figure 2b. Signal intensities were quantitated using Image J after normalization to GAPDH levels.

Supplementary Figure S3

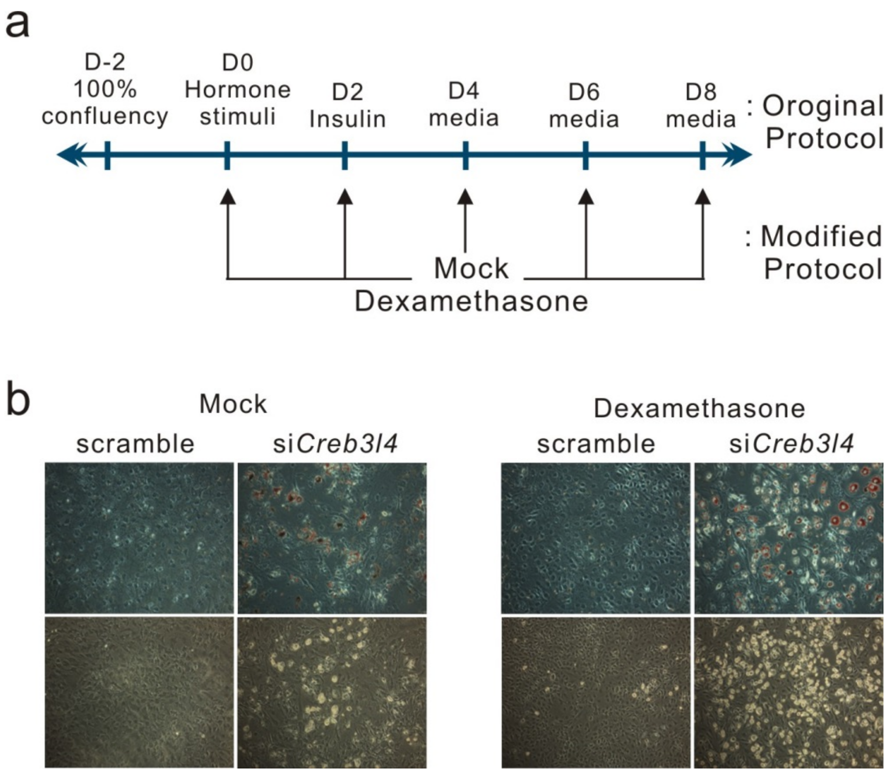

Supplementary Figure S3. Effect of *Creb3l4* ablation on adipocyte differentiation (a) A modified protocol for inducing adipocyte differentiation. Induction of adipogenesis in 3T3-L1 cells expressing si*Creb3l4*. (b) Cells were treated with either mock or dexamethasone (1 μM) in 10% FBS–DMEM. The medium was changed every 2 days. After 8 days, the cells were stained with oil-red O. Microscopic views representative of three independent experiments are shown.

Supplementary Figure S4

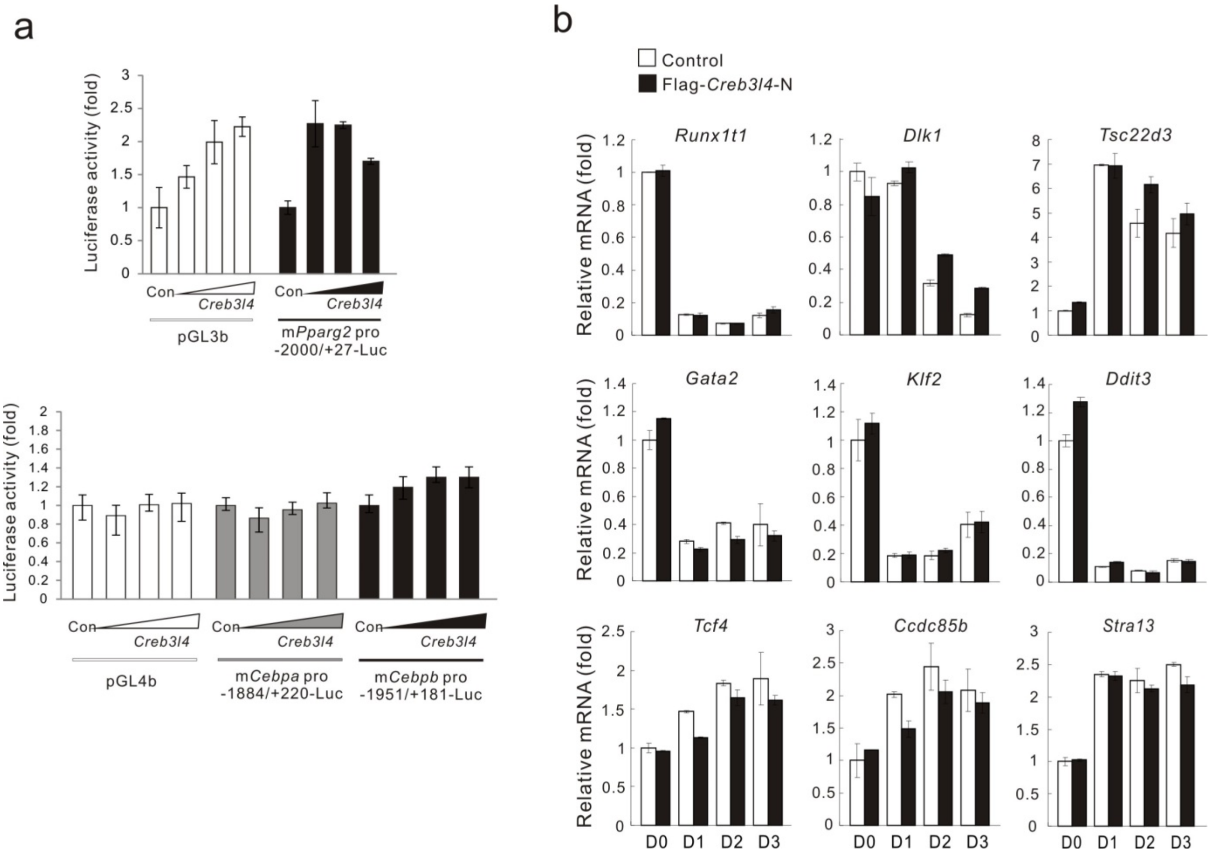

Supplementary Figure S4. Effect of CREB3L4 on the promoter activities of adipogenic factors and gene expression of negative transcriptional regulators. (a) Effect of CREB3L4 on the promoter activities of *Pparg2, Cebpa*, and *Cebpb*. NIH-3T3 cells were cotransfected with pcFlag-*Creb3l4* (0, 50, 100, or 200 ng), luciferase reporters (500 ng; mouse *Pparg2* promoter covering -2000/+27, mouse *Cebpa* promoter covering -1884/+220, mouse *Cebpb* promoter covering -1951/+181), and *Renilla* luciferase plasmid. The total amount of transfected plasmid was adjusted to 200 ng by adding empty vector. Luciferase activities were normalized to *Renilla* luciferase activities to adjust for transfection efficiency. Normalized activities are shown as the mean ± SD of three independent experiments performed in triplicate, and are expressed as fold-increase relative to the basal activity. (b) The mRNA levels of negative regulators of adipogenesis, as determined by real-time PCR. 3T3-L1 preadipocytes cells were transfected with Flag-*Creb3l4* (N) by electroporation, and differentiation was induced with medium containing DMI (0.5 mM 3-isobutyl-1-methylzanthine [IBMX; M], 1 μM dexamethasone [D], and 1 μg/ml of insulin [I]). The cells were harvested at the indicated times. Error bars represent mean ± SD.

Supplementary Figure S5

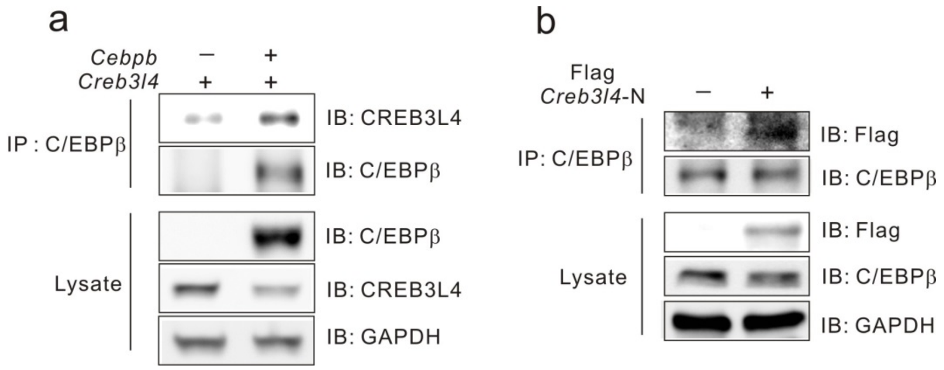

Supplementary Figure S5. Interaction between C/EBPβ and CREB3L4. (a) NIH-3T3 cells were co-transfected with *Creb3l4* and *Cebpb* expression vectors and then immunoprecipitated using an anti-C/EBPβ antibody. (b) Interaction of endogenous C/EBPβ and CREB3L4 in 3T3-L1 cells. Cells were transfected with Flag-tagged *Creb3l4*-N expression vector and precipitated with anti-C/EBPβ antibody.

Supplementary Figure S6

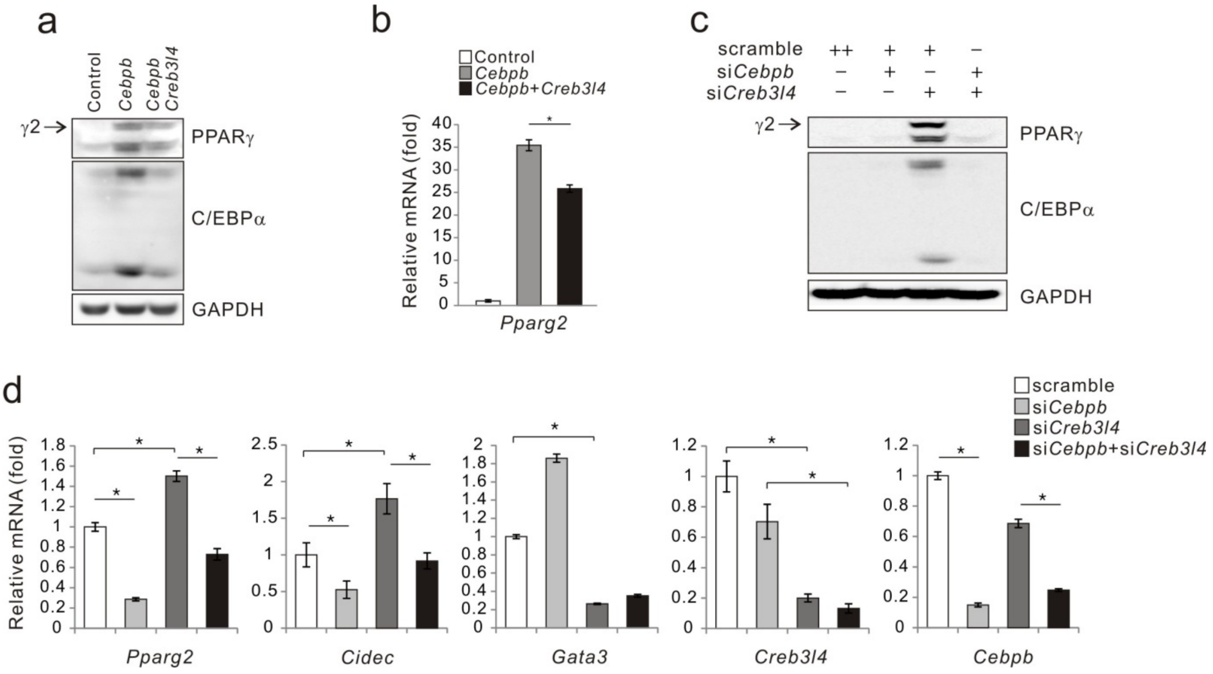

Supplementary Figure S6. Effect of C/EBPβ on si*Creb3l4*-induced PPARγ2 expression. (a) 3T3-L1 cells were transfected with *Cebpb* or *Cebpb/Creb3l4* and stimulated to differentiate with 10% FBS–DMEM containing insulin (1 μg/ml) every 2 days. Immunoblot of transcription factors involved in adipogenesis in 3T3-L1 cells at day 8. (b) 3T3-L1 cells were transfected with constructs containing *Cebpb* or *Cebpb/Creb3l4*. Expression of *Pparg2* in 3T3-L1 cells expressing *Cebpb* or *Cebpb/Creb3l4* at day 0, as analyzed by real-time PCR. (c) Immunoblot of C/EBPα and PPARγ2 at day 8. si*Cebpb* and si*Creb3l4* were transfected into 3T3-L1 cells. The cells were stimulated to differentiate with 10% FBS–DMEM containing insulin (1 μg/ml) every 2 days. (d) The mRNA levels of *Pparg2, Cidec*, *Gata3*, *Creb3l4*, and *Cebpb* in 3T3-L1 cells transfected with si*Cebpb* and si*Creb3l4* at day 0. Values are expressed as mean ± SEM, n = 3 per group, $^*P < 0.05$.

Supplementary Figure S7

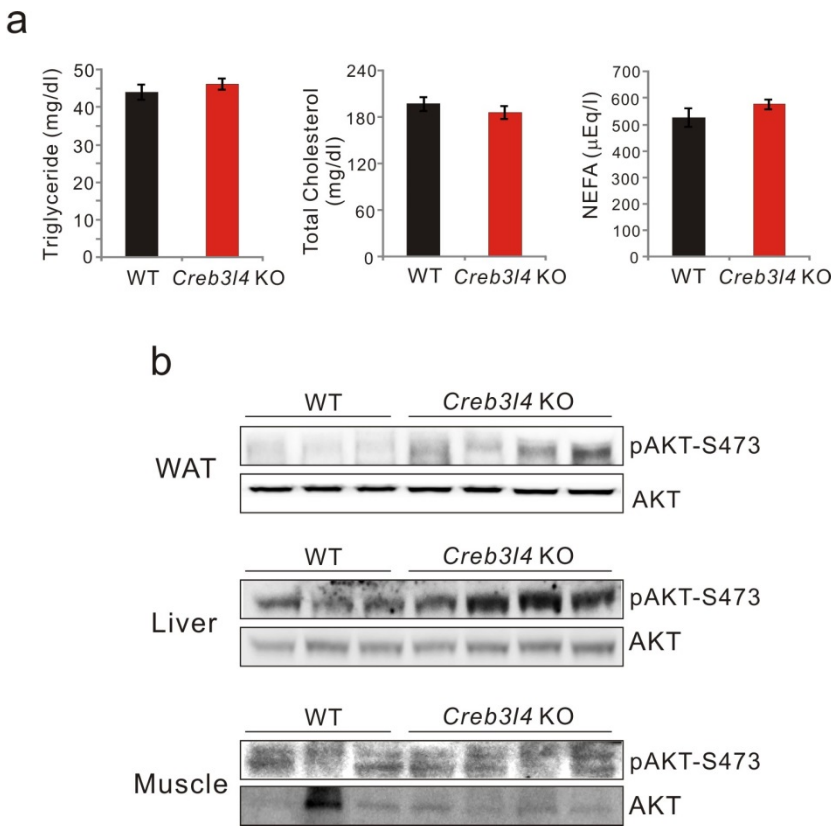

Supplementary Figure S7. The phenotype of *Creb3l4* KO mice (a) Serum lipid profiles, triglyceride, total cholesterol, and non-esterified fatty acid (NEFA) levels were measured by enzymatic methods using an autoanalyzer (n = 7–15). Values are expressed as mean ± SEM. (b) Mice fed HFD were fasted for 6hr and then received insulin (5U/kg) by intraperitoneal injection. After 5 min, the mice were sacrificed. One gram of each tissue was homogenized in RIPA buffer and subjected to western blot using anti-pAKT-S473, anti-AKT-total antibodies.

Table S1. List of primer sequence for gene-specific real-time PCR.

Gene	Forward (5′→ 3′)	Reverse (5′→ 3′)
Creb3l1	TCTTTGATGACCCTGTGCTGGA	TCCTCCATCTTGACAGGCACAA
Creb3l2	CAAGCTGAGCGAGCTGTCAGAG	CCACCTCCATTGACTCGCTCTT
Creb3l3	GACAGCATGGAGGTCCTTGA	TCCCAGGATGCAATTTAGGA
Creb3l4	ATATCTTCTCGACGGGATCCTT	TCCCTACCAGGAGATGTTTC
Pparg2	CCAGAGCATGGTGCCTTCGCT	CAGCAACCATTGGGTCAGCTC
Cebpa□	GAACAGCAACGAGTACCGGGTA	GCCATGGCCTTGACCAAGGAG
Cebpb	CAAGCTGAGCGACGAGTACA	CAGCTGCTCCACCTTCTTCT
Gata3	AACAGACCCCTGACTATGAAGAA	GGTTGAAGGAGCTGCTCTTGGG
Gata2	GGCACCTGTTGTGCAAATTGTCA	CCTTCTTCATGGTCAGTGGC
Runx1t1	GGCCAGCGGTACAGTCCAAATA	GATAGGAGTCCCTGTAGTGGTG
Dlk1	GACCCACCCTGTGACCCC	CAGGCAGCTCGTGCACCCC
Tsc22d3	GCTGCACAATTTCTCCACCT	GAGGTCCATGGCCTGCTCAAT
Klf2	GGCCCCAGCTTCGGCGGC	TGAGGAGACCGCGCGTGGCA
Ddit3	TGAGTCCCTGCCTTTCACCTTG	GGCTCCTCTGTCAGCCAAGCTA
Tcf4	AGAGATGAGAGCGAAGGTGGT	CTGTCGTTCCTTCCGGGCC
Ccdc85b	CTGATGCAGGAGGTGAATCG	CGAGTCCAGAAAGCAGCAGA
Stra13	GGAAGGAACTGGTGAGCAGAC	GCAGCCTCTAGTACGAAGATCC
Cidec	TCCAGGACATCTTGAAACTT	GGCTTGCAAGTATTCTTCTGT
Lxra	GAGAAGCTGGTGGCTGCCCA	AGCTGTAGGAAGCCAGGGAG
Adipoq	ATGGCAGAGATGGCACTCCT	CCTTCAGCTCCTGTCATTCC
Pepck	ACACACACACATGCTCACAC	ATCACCGCATAGTCTCTGAA
Rplp0	TGGCCAATAAGGTGCCAGCTGCTG	CTTGTCTCCAGTCTTTATCAGCTGCAC
